# Synthesizing Dimensions of Digital Maturity in Hospitals: Systematic Review

**DOI:** 10.2196/32994

**Published:** 2022-03-30

**Authors:** Rhona Duncan, Rebekah Eden, Leanna Woods, Ides Wong, Clair Sullivan

**Affiliations:** 1 School of Information Systems Queensland University of Technology Brisbane Australia; 2 Centre for Health Services Research The University of Queensland Herston Australia; 3 Digital Health Cooperative Research Centre Australian Government Sydney Australia; 4 Digital Health Research Network The University of Queensland Brisbane Australia; 5 Clinical Excellence Queensland Queensland Health Brisbane Australia; 6 Metro North Hospital and Health Service Brisbane Australia

**Keywords:** digital maturity, digital capability, eHealth, digital hospitals, capability model, maturity model, literature review, electronic medical records

## Abstract

**Background:**

Digital health in hospital settings is viewed as a panacea for achieving the “quadruple aim” of health care, yet the outcomes have been largely inconclusive. To optimize digital health outcomes, a strategic approach is necessary, requiring digital maturity assessments. However, current approaches to assessing digital maturity have been largely insufficient, with uncertainty surrounding the dimensions to assess.

**Objective:**

The aim of this study was to identify the current dimensions used to assess the digital maturity of hospitals.

**Methods:**

A systematic literature review was conducted of peer-reviewed literature (published before December 2020) investigating maturity models used to assess the digital maturity of hospitals. A total of 29 relevant articles were retrieved, representing 27 distinct maturity models. The articles were inductively analyzed, and the maturity model dimensions were extracted and consolidated into a maturity model framework.

**Results:**

The consolidated maturity model framework consisted of 7 dimensions: strategy; information technology capability; interoperability; governance and management; patient-centered care; people, skills, and behavior; and data analytics. These 7 dimensions can be evaluated based on 24 respective indicators.

**Conclusions:**

The maturity model framework developed for this study can be used to assess digital maturity and identify areas for improvement.

## Introduction

Planning a strategic roadmap to successful digital health transformation is challenging [1] due to a busy health landscape with competing drivers for change [2-4]. This is further compounded by the myriad of new technologies health care providers can select from to advance their digital health agenda. Despite both the rapid global uptake of eHealth technologies [5] and digital health being viewed as a panacea [6] for achieving the “quadruple aim” of health care (ie, reducing costs, improving patient experience, improving the work life of health care providers, and advancing population health) [7], the outcomes of digital health transformation are inconclusive and mixed [8,9]. One proposed method for strategically developing a digital health agenda is to follow a roadmap informed by digital maturity assessments [10,11].

In health care, digital maturity is defined as the extent to which digital systems are leveraged to provide high-quality health care, resulting in improved services and service delivery for an enhanced patient experience [12]. Assessing digital maturity is particularly important in hospital settings, due to (1) the complexity and cost of health service delivery involving multidisciplinary teams in acute, high-cost care settings [13]; (2) the necessity for rapid digital transformation that leverages eHealth technologies to cater to the needs of an aging population with increased rates of chronic disease [14]; and (3) the difficulties justifying business cases for large-scale electronic medical record system implementations, which require significant upfront and ongoing costs [15].

To assess digital maturity, a maturity model can be used to allow an organization to evaluate its current digital status across a series of dimensions [1]. However, limitations exist in current approaches for measuring digital maturity in hospitals, as there is a lack of consensus over which dimensions should be assessed [11]. Others have argued that current assessments of digital maturity are insufficient due to their primary focus on technology, with limited incorporation of organizational and human factors [4]. This is further supported by Carvalho et al [16], who emphasize that most digital maturity models lack sufficient depth and breadth for adequate assessment. Currently, there is still no agreement or convergence on how to assess digital maturity in health care.

Failure to understand the appropriate dimensions for assessing the digital maturity of hospitals will hamper the success of digital health transformation and be detrimental to health care outcomes. Therefore, a systematic literature review was conducted to find what dimensions are currently used to assess digital maturity in hospitals. As such, our aim was to synthesize the maturity model dimensions that are currently used when assessing digital hospital maturity to develop a consolidated digital maturity framework. Such a synthesis is necessary for, and will be beneficial to, health care executives and strategic decision-makers in evaluating and planning for the transformation of their practice. In addition, this synthesis will be beneficial to researchers as it consolidates the maturity dimensions and provides areas for future research to further refine and strengthen maturity models and their applications.

## Methods

A systematic literature review following the guidelines of Templier and Pare [17] was conducted in December 2020 of articles that describe how digital maturity is assessed in hospital settings. In line with these guidelines, this systematic literature review was developmental in nature, in that it sought to develop a consolidated digital maturity framework. Therefore, unlike aggregative reviews, which seek to include *all* articles relevant to the phenomenon of interest, developmental reviews seek to cover only a *sample* of articles relevant to the phenomenon of interest [17].

To extract articles, medical databases (eg, PubMed, Cochrane, and Medline) and the Association for Information Systems College of Senior Scholars’ Basket of Eight journals were searched using the following search string: (“maturity model*” OR “digital capabilit*” OR “digital maturity”). These databases and sources were selected due to the prominence of digital health in these domains. However, due to the breadth of information systems literature examining digital maturity across a myriad of contexts other than health care, the following search condition was added for a more targeted review of the information systems sources: “AND (“health” OR “healthcare”)”. This additional search condition was not added to medical databases due to their targeted focus.

Articles were excluded if they were (1) focused on settings other than hospitals, as the implementation of eHealth technologies in different contexts (eg, acute vs primary care) requires vastly different resources with large heterogeneity in impact measurement; (2) focused on maturity models not related to digital health (ie, training maturity); (3) not focused on digital maturity; (4) published in a language other than English; and (5) not a full-text article (ie, posters or extended abstracts).

**Figure 1:**
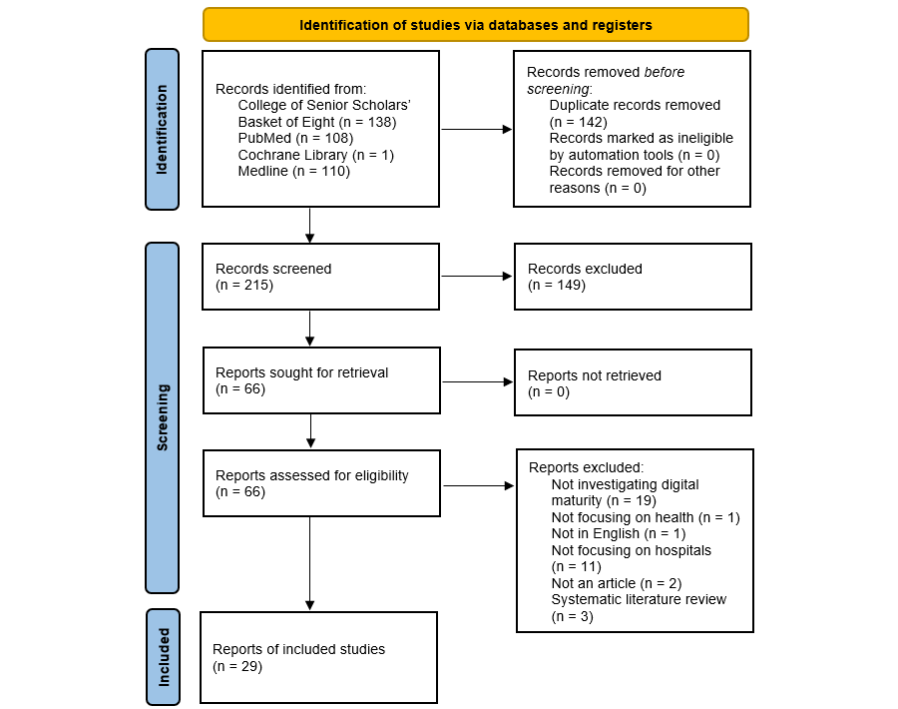
PRISMA (Preferred Reporting Items for Systematic Reviews and Meta-Analyses) flowchart.

As illustrated in Figure 1 [18], 357 articles were returned from the search, and after removing duplicates, 215 remained. Initially, the first author screened the abstracts resulting in 149 articles being removed and 66 potentially relevant articles being retained. Next, a full-text review was conducted to determine eligibility, resulting in the exclusion of 37 articles. A total of 29 articles were deemed applicable for analysis. To ensure no relevant articles were missed, backward searching of the references was performed. Consistent with Saldaña [19], intercoder corroboration was performed at each stage by the second author when determining whether an article should be included.

To analyze the relevant articles, inductive coding [20] was performed in NVivo (version 12 Plus; QSR International), with maturity model dimensions extracted. These were first extracted using verbatim codes [19] with 245 raw maturity nodes (the nodes included terms such as digital architecture, enterprise architecture, infrastructure, technology capabilities, reliability, decision support systems, picture archiving and communication system [PACS], and software applications). In some instances, the raw maturity nodes represented the specific maturity model dimensions as mentioned in the papers, while in other instances it referred to digital maturity stages, as some maturity models only provided stages rather than specific dimensions.

Through the constant comparison method [20], these raw maturity nodes were grouped into respective indicators based on commonalities. This involved considering the definition of each raw maturity node, as in some cases the name of the raw maturity node (as extracted verbatim from the paper) did not reflect its inherent meaning. By comparing the specific definitions of the raw maturity nodes, similar definitions were consolidated into a single indicator. For instance, digital architecture, enterprise architecture, and infrastructure were all related to information technology (IT), so were grouped into the IT infrastructure indicator, while technology capabilities and system reliability were grouped into the technical quality indicator, and decision support systems, PACS, and software applications were grouped into the systems and services indicator. In total, 24 indicators were identified. Following this, constant comparison was again performed to aggregate the indicators into a consolidated set of dimensions based on commonalities amongst indicators. For instance, the IT infrastructure, technical quality, and systems and services indicators were grouped into the IT capability dimension. In total, 7 dimensions were identified.

To provide further confidence in our findings, reliability assessments were also performed. First, coder corroboration was conducted. The first author independently performed coding of verbatim measures (ie, raw maturity nodes) to indicators, then grouped the indicators into dimensions and discussed these decisions with the second author until consensus was reached [19]. This involved ensuring that all verbatim measures were accurately mapped to the indicators. Through discussion, some of the verbatim measures were moved to a different indicator to better reflect their underlying definitions. Subsequently, additional coder corroboration of the indicators and dimensions was performed. This involved the first 2 authors discussing the indicators and dimensions with the rest of the authorship team and resulted in updates to some of the names and definitions [19]. Second, external reliability checks were performed and the dimensions were discussed at external forums, including (1) a statewide digital health steering committee in April 2021, attended by 14 members, and (2) the statewide “Digital Health Grand Rounds” in May 2021, attended by 120 health service executives, digital health researchers, and clinicians. Consensus was reached at the forums on the derived dimensions.

## Results

### Digital Maturity Dimensions

In total, 27 distinct maturity models were examined (Multimedia Appendix 1). In some instances, multiple maturity models were examined in a single paper, and in other instances, the same maturity model was examined in multiple papers. In total, 14 papers validated an existing maturity model, 10 papers proposed a new maturity model but did not validate the maturity model, 4 papers both proposed and validated a new maturity model, and 1 paper extended an existing maturity model.

Overall, 24 indicators were identified, which were consolidated into 7 digital maturity dimensions: strategy; IT capability; interoperability; governance and management; patient-centered care; people, skills, and behavior; and data analytics. The dimensions are described in Table 1; detailed examples of the indicators are provided with examples of their verbatim measures in Multimedia Appendix 2. Multimedia Appendix 3 illustrates how the distinct digital maturity models mapped onto the 7 digital maturity dimensions.

The findings of this review identified that digital maturity is predominantly assessed based on management-oriented dimensions and technology-related dimensions. Governance and management (n=22 articles) has been the most prevalent dimension of digital maturity, followed by IT capability (n=18), people, skills, and behavior (n=17), interoperability (n=15), and strategy (n=14). Comparatively, limited research has examined data analytics (n=6) and patient-centered care (n=3). Each dimension is further described in the below subsections.

**Table 1:** Description of the Digital Maturity Dimensions.

Dimension	Description	Indicators
Strategy	The extent to which the organization has developed and implemented a strategic plan to achieve its goals and objectives [16]	Strategic adaptability, strategic alignment, strategic focus
Information technology capability	The extent to which the organization has adopted and implemented information technology infrastructure, digital systems, technologies, and services [21] that are usable and effective [22]	Information technology infrastructure, technical quality, systems and services
Interoperability	The extent to which data and information can be exchanged between systems within the organization, across care settings, and with patients, caregivers, and families [11]	External interoperability, internal interoperability, semantic interoperability, syntactic interoperability
Governance and management	The extent to which the organization embraces leadership, policies and procedures, structures, risk management of quality and safety, integrated workflows, relationship building, and capacity building [23]	Change management, data governance, leadership and management, risk management, standards, cultural values
Patient-centered care	The extent to which patients, caregivers, and families can actively participate in their health decisions, have access to information and health data, and cocreate services and service delivery [24]	Patient empowerment, patient focus
People, skills, and behavior	The extent to which stakeholders (internal and external) are digitally literate and motivated to leverage technology [11,25]	Education and training, knowledge management, individual competence, technology usage
Data analytics	The extent to which the organization uses data for effective decision-making for the organization, patients, and population health [1]	Descriptive analytics, predictive analytics

### Governance and Management

The governance and management dimension is described as the extent to which the health care organization possesses formalized and committed leadership, as well as formalized policies, procedures, structures, and workflows [23]. Six indicators comprise the governance and management dimension: leadership and management, change management, cultural values, standards, risk management, and data governance.

The leadership and management indicator encompasses the executive team’s commitment to and support for improving clinical quality [26-28] and fostering innovation across the hospital [24]. This support is essential for all levels of the workforce. The change management indicator recognizes the need to encourage individuals to embrace planned change to achieve desired outcomes [11,29]. The need for innovation and embracing change is further evident in the cultural values indicator, which espouses values of encouraging innovative behaviors [11,29] within a trusting [26-28] and inclusive environment [30,31]. The standards indicator assesses the extent to which processes [30-32], policies, and procedures are based on the standards that have been formally agreed and mandated [24] and the extent to which these contribute to optimizing the health care organization [11,12,33]; nevertheless, this indicator is not contrary to innovation. The risk management indicator acknowledges the need for the workforce to identify, mitigate, and report risks to ensure the safety, security, and privacy of patients [1,11,26-28] and the workforce [21]. The data governance indicator further assesses whether data integrity, security, and privacy are preserved across the digital systems in health care settings [12], supported by standardized processes and protocols for accessibility and authorization [22,23,34].

### IT Capability

The IT capability dimension represents the extent to which the organization has implemented IT infrastructure, digital systems, technologies, and services [21] that are usable and effective [22]. This dimension comprised three indicators: systems and services, IT infrastructure, and technical quality.

The systems and services indicator, which examines the digital systems implemented to support clinical care, is the most prominent indicator within this dimension. The systems identified as being important to digital maturity include electronic medical records, clinical decision support systems, e-prescribing, PACS [11,35,36], orders and results management, asset and resource optimization systems [22], and remote and assistive care systems [1,21]. The IT infrastructure indicator focuses on infrastructure [21,37] and architecture [1] designed and installed to support the aforementioned systems and services [12]. The systems and services indicator, as well as the IT infrastructure indicator, largely examine what technology and supporting structures have been implemented but do not account for their effectiveness. Alternatively, the technical quality indicator focuses on how effective, efficient, and fit for purpose the digital systems are [21,38].

### People, Skills, and Behavior

The people, skills, and behavior dimension assesses the extent to which stakeholders, both internal and external to the health care organization, are digitally literate and motivated to leverage digital health systems [11,25]. This dimension consists of four indicators: education and training, knowledge management, individual competence, and technology usage.

The education and training indicator relates to the strategies adopted by the health care organization to provide individuals with opportunities to grow and develop clinical and technical skills, as well as collaboration and teamwork skills [16,29]. The knowledge management indicator refers to the extent to which workforce capability grows through creating, managing, and sharing knowledge [23,30]. These two indicators focus on ensuring organizational practices are in place to foster skill development and knowledge acquisition, whereas the individual competence and technology usage indicators focus on the actual skill sets and behaviors of individuals. For instance, at the individual level, the individual competence indicator takes into consideration that individuals need to possess skills, knowledge, and capability to use digital systems [21]. In contrast, the technology usage indicator recognizes that systems can be used in different ways and that digitally mature organizations need to ensure systems are used as intended [12] in a pervasive and consistent manner [26-28].

### Interoperability

The interoperability dimension represents the extent to which data and information can be exchanged between systems within the organization, across care settings, and with patients, caregivers, and families [11]. Four interoperability indicators were identified: external interoperability, internal interoperability, semantic interoperability, and syntactic interoperability. The former 2 indicators relate to how information is exchanged between different actors within and between organizations (ie, intraorganizational vs interorganizational information exchange). The latter 2 indicators relate to data transformation and distinguish between the technical and meaningful structure of the information exchanged.

The external interoperability indicator assesses the adoption of standards to integrate systems, services, and data across the entire health care system [1,11,30,39-41]. Conversely, the internal interoperability indicator assesses the integration of systems and data across departments within a single health care organization [30,40]. The external interoperability indicator was more prevalent in the literature than the internal interoperability indicator.

The semantic interoperability indicator examines the extent to which information exchanged between digital systems can be accurately interpreted and understood by each system involved [34,42]. As such, the semantic interoperability indicator is dependent on the transparency of the underlying lexicon and data dictionary to ensure the intended meaning of the information exchange is retained [42]. Alternatively, the syntactic interoperability indicator represents the extent to which technical standards have been defined to enable the consistent, effective, and efficient integration of digital systems and services [30,34].

### Strategy

The strategy dimension represents the extent to which the organization has developed and implemented a strategic plan to achieve its goals and objectives [16]. The strategy dimension includes three indicators: strategic focus, strategic alignment, and strategic adaptability. This dimension is built on the premise that the digital strategy and the organizational strategy should be aligned and adaptable to support the accomplishment of measurable goals and outcomes related to quality and safety [26-28].

The strategic focus indicator was the most prevalent, with an emphasis on quality and safety [26-28], sustainability and cost effectiveness [39], and ensuring the systematic evaluation of quantifiable results and objectives [24,26-28]. While the strategic focus indicator centers on the core elements that health care organizations focus on, the strategic alignment indicator details the need for the digital strategy to be aligned with the organizational strategy [12,43]. To accomplish this, the digital strategy needs to be grounded on clinical benefits and outcomes [1,11]. In contrast, the strategic adaptability indicator recognizes the importance of organizational strategy and digital system dynamism [1,11,16] and that both should be capable of responding to environmental challenges [44] and emerging opportunities [11,16,24].

### Data Analytics

The data analytics dimension examines the extent to which the organization uses data collected in its digital systems for effective decision-making to benefit the organization, patients, and population health [1]. Few studies have reported on this dimension and as such only 2 indicators have been identified: descriptive analytics and predictive analytics.

The descriptive analytics indicator is the extent to which data is analyzed to identify and understand historical patterns and trends, facilitating effective decision-making [1]. The predictive analytics indicator focuses on the analysis of data that enables future potential risks and opportunities to be identified to aid decision-making [24], including “proactive/predictive models of care” [11].

### Patient-Centered Care

The patient-centered care dimension encompasses the extent to which patients, caregivers, and families can actively participate in their health decisions, access information and their health data, and cocreate services and service delivery [24]. Only 3 articles in this review examined patient-centered care as a dimension of digital maturity, which resulted in 2 indicators: patient focus and patient empowerment.

The patient focus indicator assesses the extent to which the role of the patient is considered, involved, and valued when designing new models of care [25,31]. The patient empowerment indicator represents the extent to which patients are encouraged to actively participate in their health decisions and have access to relevant information and health data [24].

## Discussion

### Summary of Key Findings

**Figure 2:**
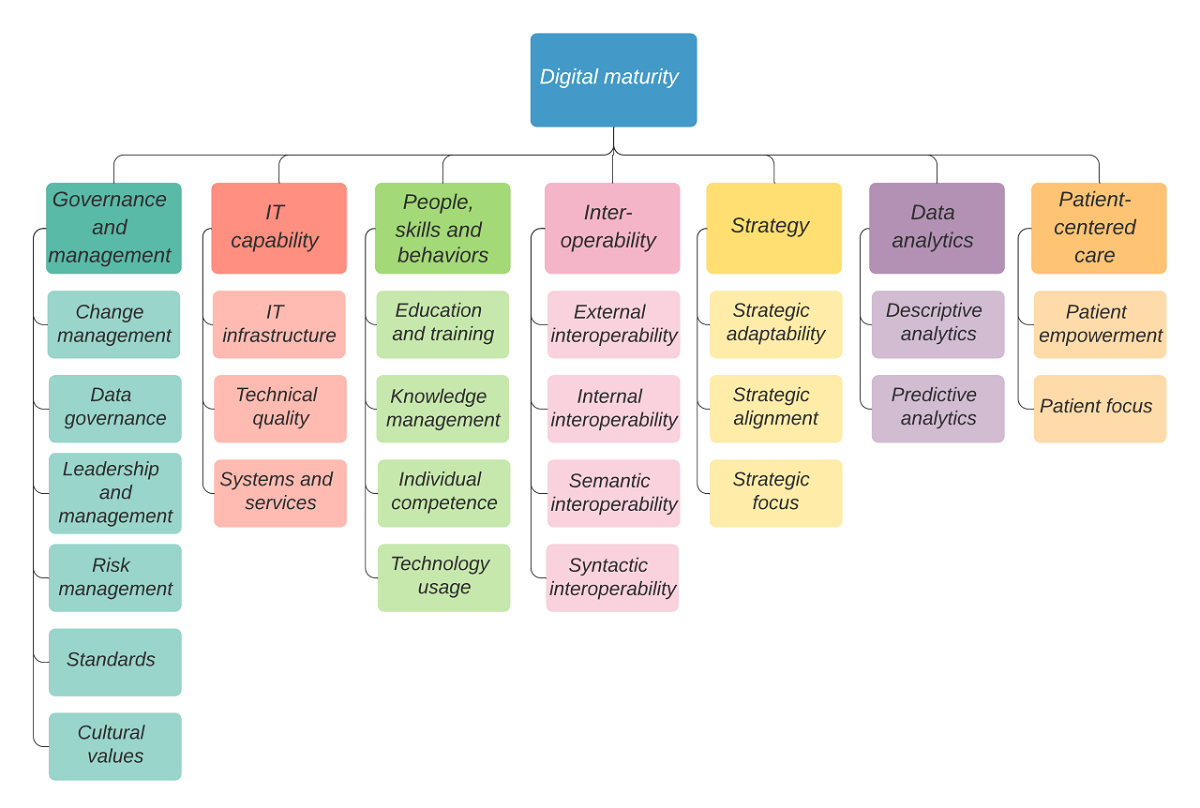
Consolidated Digital Maturity Model Framework for Hospitals.

In summary, we identified 7 dimensions (ie, strategy; governance and management; IT capability; interoperability; data analytics; people, skills, and behavior; and patient-centered care) of digital health maturity that hospitals need to consider when strategically planning their digital health agenda. In addition, we identified 24 indicators that can be used to measure these dimensions (Figure 2). To operationalize these indicators, future research should seek to rigorously develop specific measurement items and follow extensive internal and external validity and reliability assessment [45].

These dimensions have received varying attention in the literature; however, as we argue in the “Implications for Future Work” section of this paper, a robust digital health maturity assessment must consider all dimensions to a sufficient depth. As such, we considered these dimensions to be equally weighted. Failure to consider a dimension could ultimately prove detrimental to the overall digital transformation agenda.

Our findings extend previous systematic literature reviews on digital health maturity models in 3 ways. First, past reviews have sought to identify various maturity models used in health care and analyze them in isolation. For instance, Carvalho et al [37] examined 14 maturity models and Gomes and Romão [46] investigated 26 maturity models commonly employed in health care, providing a descriptive account of each. In contrast, this study synthesized the dimensions present across maturity models to derive a consolidated framework (Figure 2). Second, other reviews have investigated maturity model dimensions, yet had aggregate dimensions that were inappropriately broad. For instance, Tarhan et al [47] developed a consolidated list of only four maturity model dimensions: business process, technology, people, and other. Their business process dimension incorporated government regulations, their technology dimension was aligned with the IT capability dimension identified in this study, and their people dimension focused largely on patient safety culture and therefore differed from the people, skills, and behavior dimension identified in this study, which examined individual-level and organizational-level factors. The “other” category incorporated a wide range of factors including “culture, strategy, governance, leadership, interoperability, and data” [47]. The maturity model framework developed in this paper provides a more granular account of factors in the “other” category represented in the previously reported dimension. Such a granular account is necessary for effective assessment.

### Implications for Future Work

Through performing this analysis, we have identified four important areas that future research should focus on: (1) balancing digital maturity dimensions; (2) evaluating the impact of dimensions on the quadruple aim of health care; (3) examining the interrelationships between dimensions; and (4) evaluating longitudinal variations in digital maturity. These are discussed in turn below.

#### Balancing Digital Maturity Dimensions

No maturity model in our review encompassed all 7 dimensions of our consolidated digital maturity model framework. The vast majority of studies focused on organizational capability (ie, governance and management and strategy), technological capability (ie, IT capability and interoperability), and individual capability (ie, people, skills, and behavior). Only 3 papers recognized patient-centered care as a dimension of digital maturity, which lags behind the goals of current medical practice. This marked difference in attention to the dimensions of the maturity models illustrates the traditional corporate focus on technical and regulatory components of digital health and the neglect of patient outcomes in the digital transformation of health care. This is a clear oversight in current digital maturity assessments, as government policies are increasingly placing the patient “at the heart of their own treatment plans so that they might develop a commitment to self-management” [48].

Moreover, while technology capability has been a prominent theme in both the IT capability and interoperability dimensions, there has been less attention paid to understanding the data analytics dimension [16]. In terms of data analytics, many of the professed benefits of digital health emanate from the “promise and potential” of the secondary use of health care data [49,50]. This capability is, further, central to prior government agendas promoting meaningful use of technology [51]. As such, future research needs to address data analytics as a key aspect of digital maturity, examining not only descriptive and predictive analytics but also the potential of prescriptive analytics.

Our findings (detailed in Multimedia Appendix 1) demonstrate that the vast majority of maturity models have been assessed in developed countries, such as the United States and the United Kingdom. Seldom is the digital maturity of hospitals in developing countries assessed (notable exceptions are the work of Yarmohammadian et al [52] and Moradi et al [31], who examined maturity models in Iran), and cross-cultural comparisons are largely overlooked. Future research therefore needs to examine the extent to which maturity models are equally applicable across cultures and settings. Ammenwerth et al [53] provide some useful guidance into how best to do so.

#### Evaluating the Impact of Digital Maturity Dimensions

While the importance of the 7 identified dimensions has been raised across multiple papers, their impact on outcomes such as the quadruple aim of health care (ie, reducing costs, improving patient experience, improving the work life of health care providers, and advancing population health [7]) has largely not been assessed (details are shown in Multimedia Appendix 4). Only 2 papers in our study triangulated digital maturity with outcomes. For instance, van Poelgeest et al [44] identified that the higher the digital maturity based on the Electronic Medical Record Adoption Model (EMRAM), the shorter the length of stay, although this was dependent on the location of the hospital. Conversely, Martin et al [12] identified that digital maturity based on a clinical digital maturity index did not influence the mortality, readmission, or complications encountered in the hospital, but found that maturity significantly improved length of stay and the number of harm-free patient care episodes.

Understanding how digital maturity influences outcomes is essential, as past research has found mixed results when assessing the outcomes of the digital transformation of health care, with recommendations made to policy makers to “identify and support the drivers of successful [eHealth] outcomes” [8]. If designed and applied correctly, digital maturity assessments could equip policy makers with tools to evaluate whether they have the drivers in place for successful digital transformation [54]. However, validation of the digital maturity dimensions is still required. Such validation will need to extend beyond measuring operational improvements such as cost savings and productivity goals and consider all 4 health care aims. Failure to adequately recognize the health care aim of improving the working conditions of health care providers will limit successful digital transformation, as demonstrated in many reports of staff dissatisfaction and burnout associated with digital technology in health care [55].

Similar concerns surrounding the validity of digital maturity models have been observed by Thordsen et al [56]. To validate the digital maturity of hospitals, it is necessary to analyze digital maturity through the lens of balanced health care outcomes, as outlined in the quadruple aim. We encourage future research using a multiple case study design to evaluate both the digital maturity dimensions and key performance indicators related to each aim and to assess whether digital maturity correlates with health care outcomes. Confounders will likely be present, but this is a necessary first step to provide evidence to health care executives regarding the need to evaluate and improve digital maturity. In addition, future research should seek to perform an intervention study with targeted improvements within each digital maturity dimension and assess the impact on the health care aims to further understand the mechanisms behind the purported relationship.

#### Understanding the Interrelationships Between Dimensions

The digital maturity dimensions in the literature reviewed here were largely examined in a subjective manner, with the dependencies and interrelationships open to interpretation, assumptions, and variability. Future research should seek to delve into these interrelationships further, as this could provide insights into the order in which hospitals should seek to improve the digital maturity dimensions. For instance, efforts to improve data analytics and IT capabilities through implementing artificial intelligence algorithms for complex clinical care may be hampered if there is no appropriate clinical governance or a clinical informatics workforce. As such, future research should seek to examine exemplar cases which have excelled in each of the dimensions to identify their drivers.

#### Conducting a Longitudinal Analysis of Dimensions

At different stages of a hospital’s digital health journey, different maturity model dimensions may need to be assessed. This is because digital systems and organizations are dynamic and, therefore, change over time. Although some maturity models decompose digital maturity into stages, these are often simple in nature. Some notable maturity models have taken this level of detail into account [16] by considering the varying measurement criteria between the different stages of maturity. But as a whole this approach has mostly been overlooked. As such, scholars should seek to perform a longitudinal investigation of digital maturity to ensure appropriate assessments are performed depending on the level of IT capability within the hospital.

### Limitations

This review is scoped to the digital maturity of hospitals and not to other health care settings. This is necessary because of the vast differences between acute health care settings and primary care. Future research should seek to investigate the digital maturity of primary care settings to identify maturity dimensions necessary for their successful transformation.

Although maturity models are widely being used in hospitals globally, it is important to note that digital maturity assessments are just one approach to planning and evaluating digital health transformations. Future research should compare the efficacy of digital maturity assessments with other approaches, for instance plans for digital health transformation benchmarking [53], the NASSS (nonadoption, abandonment, scale-up, spread, and sustainability) framework [57], and organizational readiness surveys [58]. Alternatively, from an evaluation perspective, organizations can adopt the measures from evaluation frameworks [59].

In addition, this literature review has been scoped to peer-reviewed outlets in medical databases and leading information systems journals, with “grey” literature excluded, which could have led to publication bias. Although this scoping may have missed some articles, it was necessary to ensure only high-quality, theoretical, rigorously developed models were included. In addition, proprietary maturity models that are used in practice but not examined in the peer-reviewed literature in this study were omitted. Nevertheless, many proprietary maturity models have been examined in peer-reviewed journals and were therefore included in this study, such as the EMRAM of the Health Information Management Systems Society.

### Conclusions

This systematic literature review resulted in the development of a consolidated digital maturity model framework consisting of 7 core dimensions and 24 indicators of digital health maturity. Future research needs to be conducted to understand how these dimensions relate to outcomes across the quadruple aim of health care, and to extend the traditional IT and corporate focus to include patient and staff considerations. In that way, digital health strategic plans will become aligned to the strategic aims of hospitals and focused on delivering the quadruple aim of health care.

## Appendix

Multimedia Appendix 1 Distinct Maturity Models.

Multimedia Appendix 2 Digital Maturity Dimensions and Indicators.

Multimedia Appendix 3 Mapping of Dimensions to Maturity Models.

Multimedia Appendix 4 Method Applied for Validated Maturity Model.

## Abbreviations

EMRAM: Electronic Medical Record Adoption Model
IT: information technology
MM: maturity model
NASSS: nonadoption, abandonment, scale-up, spread, and sustainability
PACS: picture archiving and communication system
