# Supplementary material for: Synthesizing Dimensions of Digital Maturity in Hospitals: Systematic Review
Source: J Med Internet Res. 2022 Mar 30;24(3):e32994. doi: 10.2196/32994 (PMC9008527; doi:10.2196/32994)
Supplement: Multimedia Appendix 1 [file jmir_v24i3e32994_app1.docx]

**Multimedia Appendix 1.** Distinct Maturity Models.

| **Maturity Model (MM)** | **Description** | **Dimensions / Capabilities** | **No. of Stages** | **Proposed new** | **Proposed Extension** | **Validated / Applied** | **Country Applied** | **Ref.** |  |
| --- | --- | --- | --- | --- | --- | --- | --- | --- | --- |
| Picture Archiving and Communication Systems (PACS) maturity model (PMM) | Examines radiology systems and electronic records in hospitals based on the extent of integration of three trends. | 3 trends (Radiology & hospital-wide process improvement; Integration optimization & innovation; Enterprise PACS & electronic records) | 5 | X |  |  |  | [59] |  |
| Extended PMM | Extends PMM to include strategic perspectives of the hospitals growth paths. | 4 strategic perspectives (Strategy execution; Technology potential; Competitive potential; Service level) | 5 |  | X |  |  | [42] |  |
| MM for Enterprise Interoperability model (MMEI) | Investigates the maturity of enterprise interoperability as the essential basis for an adaptive org. | 3 aspects (Conceptual interoperability (semantic); Technical interoperability; Organizational interoperability) | 5 |  |  | X | Luxem-bourg | [34] |  |
| Healthcare Info and Mgmt Systems Society (HIMSS) Health Usability MM | Assesses the value provided through the usability of IS across health org. | 5 dimensions (Focus on: users; Mgmt; Process & infrastructure; Resources; Education) | 5 | X |  |  |  | [33] |  |
| People Capability MM (PCMM) | Evaluates the maturity of the capability of people in an org. | 4 process areas (Developing individual capability; Building workgroups & culture; Motivating & managing performance; Shaping the workforce). | 5 |  |  | X | Iran | [51] |  |
| Electronic and Comp Clinical Prediction Rules MM (eCPR MM) | Evaluates how mature an org is in developing a learning health system through evidence-based medicine in the form of clinical prediction rules. | 4 interoperability layers (Syntax; Semantics; Transport; Services) | 6 | X |  |  |  | [41] |  |
| QMS & ISO 10014 Standard | Assesses the digital maturity of hospitals with respect to quality mgmt systems | 8 dimensions of QMS are combined with the Plan-Do-Check-Act cycle | 5 |  |  | X | Iran | [31] |  |
| High Reliability Health Care MM (HRHCM model) | Considers embedding three domains to ensure safety is continuously and sustainably improved. | 3 domains divided into 14 areas of performance (Leadership; Safety culture; Robust process improvement) | 4 |  |  | X  X  X | USA | [26-28] |  |
| Hospital Business-IT alignment (H-BIT) | Assesses strategic alignment of the IT and business architectures in hospitals | 4 building blocks (Link strategy to value activities; Identify stakeholder-specific strategy; EA capability modelling; EA capability ast) via 5 levels of adoption | 5 | X |  |  |  | [30] |  |
| Hospital Supply Resource Mgmt (HSRM) | Assesses how effective and reliable the procedures of a hospital’s supply resource mgmt system are | Combination of 3 domain specific dimensions (Strategy; Tactics; Operations) with 3 maturity dimensions (Objects, Processes; People) |  | X |  |  |  | [30] |  |
| Hospital Coop MM (HCMM) | Assesses how well structures and processes within a hospital and between hospitals enable cooperation. | 3 dimensions (Strategic layer; Organizational layer; Info layer) with 12 reference points or items each | 4 | X |  |  |  | [30] |  |
| Patient-centric frame-work | The patient-centric framework focuses on patients through assessing the digital maturity of an entire care network across four themes. | 4 themes (Resource availability & Ability/Capacity to use; Usage; Interoperability; Impact) | 4 | X |  |  |  | [25] |  |
| Healthcare Game MM | Assesses the extent hospital use gamification to enhance staff training and patient experience. | 4 perspectives (Value; Process; Coverage; Type) | 5 |  | X | X | The Nether-lands | [32] |  |
| NHS' Digital Maturity Ast. (DMA) | Self-ast. tool focusing on the ability of the org to integrate its IS internally and across the broader healthcare network for improved patient-centric services. | 3 themes (Readiness; Capability; Infrastructure) | N/A |  |  | X | UK | [1] |  |
| HIMSS Electronic Medical Record Adoption Model (EMRAM) | Determines the maturity of electronic medical record adoption across the org. | 3 areas of factors (Environmental; Organizational; ICT) | 8 |  | X | X  X | The Nether-lands  Turkey | [11, 35, 43] |  |
| SCIROCCOB3-MM | Assesses 12 dimensions essential in the mgmt of mature integrated care delivery. | 12 dimensions with 30 indicators (Capacity building; Readiness to change; Structure & governance; Info & eHealth services; Finance & funding; Standardization & simplification; Removal of inhibitors; Population approach; Citizen empowerment; Evaluation methods; Breadth of ambition; Innovation mgmt) | 6 |  |  | X | 12 European countries | [24] |  |
| British Virgin Islands’ NHS IS for Health MM (IS4H-MM) | Measures four strategic areas relevant to public health policy and knowledge mgmt, based on the strategy of “informed decision-making for better health outcomes” [23] | 4 strategic areas (Mgmt & governance; Knowledge mgmt & sharing; Innovation & performance; Data mgmt & IT) | 5 |  | X | X | Virgin Islands | [23] |  |
| Health IS MM (HISMM) | HISMM presents the 6 influencing factors in a grid format ast. framework. It identifies the characteristics per factor for each of the 6 stages and aims to cater for the complexity of healthcare. | 6 influencing factors comprising critical variables (Data analysis; Strategy; People; Electronic medical record; IS & IT infrastructure) | 6 | X  X  X |  |  |  | [16, 21] |  |
| Infra-structure MM (IMM) | Evaluates how value can be produced for the org through info access and process optimization. | 5 technology domains (Reliability; Availability; Usability; Relevance; Completeness) | 8 | X |  |  |  | [37] |  |
| Interoper-ability MM (IntMM) | Measures the level of interaction between public services from a digital service perspective | 3 domains (Service delivery; Service consumption; Service mgmt) | 5 |  |  | X | Greece | [39] |  |
| Clinical Decision Support MM (CDS MM) | Assesses an orgs CDS capabilities through 3 pillars to assist orgs to achieve improved outcomes through effective CDS use | 3 “pillars” (Content creation; Analytics & reporting; Governance & mgmt) with 3 capabilities (Learning health system; Interorganizational benchmarking & sharing; Proactive opportunity discovery) | 5 | X |  | X | USA | [40] |  |
| NHSs Clinical Digital Maturity Index (CDMI) | Self-ast. tool of an extensive ast. framework to assess the overall digital maturity of a hospital. | 3 themes (Readiness; Capability; Infrastructure) with 13 sections | Scale: 0-1400 |  |  | X  X | UK  UK | [12, 22] |  |
| Patient Real Time Feedback (RTF) maturity | Evaluates the maturity of real time feedback from patients, which is closely linked to the digital maturity of the org. | 4 domains (Capacity/Resource; Usage; Interoperability; Impact) | Score: 0-4 |  |  | X | UK | [38] |  |
| NHSs Digital excellence ast. | Combines NHS’s DMI with HIMSS EMRAM to evaluate the technology and organizational capabilities to examine how hospitals are connected to the broader health network. | 6 capabilities (Technological within hospitals; Technological - communication with other parts of the health & social care system; Technological - communication with patients & carers; Org. culture; Workforce; Strategy | N/A | X |  |  |  | [11] |  |
| NHSs Digital Maturity Indicator (DMI) | Self-ast. Tool that is a refinement of the DMA and CDMI MM. | 3 themes (Readiness; Capabilities; Infrastructure) with 14 sections | N/A |  | X |  |  | [11] |  |
| Health IT Safety Measure-ment (HITS) Frame-work | Based on the SAFER (Safety Assurance Factors for EHR Resilience) guide to measure, monitor, and improve the safety of health IT use in hospitals. | 3 domains with 6 principles (Safe Health IT (Data availability; Data integrity; Data confidentiality); Using Health IT (Complete/correct health IT use; Health IT system usability); Monitoring Safety (Surveillance & optimization)) | N/A |  |  | X | UK | [22] |  |
| Person-Centered Digital Healthcare (PCDHc) Frame-work | Evaluates digital capabilities required by healthcare professionals to deliver person-centered digital healthcare | 4 themes with 13 categories (Change mgmt (Professionalism; Education; Professional standards; Non-technology skills); User application (Use development; Holistic care; Partnership); Data, info & knowledge (Technology skills; Technology literacy; Managing technology); Innovation (Innovative practice; Innovative behavior; Applied innovation)) | N/A | X |  |  |  | [29] |  |
| ^a^info: information; mgmt: management; comp: computable; coop: cooperation; ast.: assessment; Ref: reference; org: organization; | | | | | | | | | |
