# Supplementary material for: Synthesizing Dimensions of Digital Maturity in Hospitals: Systematic Review
Source: J Med Internet Res. 2022 Mar 30;24(3):e32994. doi: 10.2196/32994 (PMC9008527; doi:10.2196/32994)
Supplement: Multimedia Appendix 2 [file jmir_v24i3e32994_app2.docx]

**Multimedia Appendix 2.** Digital Maturity Dimensions and Indicators.

| **Dimension** | **Indicator** | **Description** | **Examples** | **Ref.** |
| --- | --- | --- | --- | --- |
| Governance and Management | Change management | The extent to which approaches are adopted to manage the individuals in embracing the proposed changes to the organization | Change management, Organizational culture, Building workgroups and culture | [11, 12, 29, 31, 51] |
|  | Data governance | The extent to which data and information are managed to ensure the factors of responsibility, authority, confidentiality, integrity, and governance are supported | Organizational interoperability, Data management and information technologies, Information governance, Data confidentiality, Data integrity | [11, 12, 22, 23, 34] |
|  | Leadership and management | The extent to which the organization manages and leads digital transformation, safety, clinical quality improvement efforts, and resource and performance | Factual approach to decision making, Governance and management, Innovation management, Leadership and culture, Organizational readiness, System approach to management, Cooperation, Motivating and managing performance, Resourcing | [11, 12, 21-24, 31, 33, 40, 43, 51] |
|  | Risk management | The extent to which the workforce identifies, mitigates, and reports risks in the organization to ensure the safety, security, and privacy of information | Information security, Surveillance and optimization, Privacy and security, Strengthening systems, Identifying unsafe conditions | [1, 11, 16, 21, 26-28, 37] |
|  | Standards | The extent to which standards and structures in the organization have been mandated, agreed upon, and contribute to optimizations | Asset and resource optimization, Governance, Standardization and simplification, Standards, Structure and governance, Process and infrastructure, Process approach | [11, 12, 24, 30-33, 59] |
|  | Cultural values | The extent to which the workforce of the organization possesses a participatory culture, which values innovation and inclusiveness | Accountability, Innovation, Organizational culture, Leadership and culture, People tactics, Trust, Employee involvement | [1, 12, 26-31, 43] |
| IT Capability | IT infrastructure | The extent to which the hardware, network resources and services have been designed, mapped, and implemented to support the required digital capabilities of the organization | Digital architecture, EA capability modelling, Infrastructure, Systems and IT infrastructure | [1, 11, 12, 16, 21, 22, 30] |
|  | Technical quality | The extent to which digital systems and technologies possess system quality and information quality | Information completeness, relevancy, reliability and usability; Medication safety; Technological capabilities within a hospital; Usage; Health IT system usability; Focus on users; Usability | [11, 21, 22, 33, 35, 37, 38] |
|  | Systems and services | The extent to which digital systems and services have been adopted and implemented | Capability, Decision support, Electronic ordering, EMR, Image management systems, IT, Medicines management and optimization, mHealth, Object operations, Orders and results management, PACS, Software applications, Systems and IT infrastructure, Telehealth, Telemedicine | [1, 11, 12, 16, 21, 22, 25-28, 30, 31, 35, 40, 41] |
| People, Skills and Behaviors | Education and training | The extent to which growth and development strategies are implemented to train and educate the workforce and patients | Developing individual capability, People, Teaching status, Training, Education | [16, 26-29, 33, 43, 51] |
|  | Knowledge management | The extent to which the workforce enhances its proficiency through creating, managing, and sharing knowledge | Capacity building, Knowledge management and sharing, People operations | [23, 24, 29, 30] |
|  | Individual competence | The extent to which the workforce, patients, and extended care network of the organization are digitally competent | Capacity/Resource, People, Shaping the workforce, Workforce | [11, 21, 25, 38, 51] |
|  | Technology Usage | The extent to which digital systems are used as intended | Software usage, Spread, Complete/correct health IT use, Usage | [22, 25-28, 35] |
| Interoperability | External interoperability | The ability of the organization to exchange data and information with external partners, enabling communication across all care settings, and with patients and caregivers | Information availability, Breadth of ambition, Integrated care models, Interorganizational benchmarking and sharing, Data availability, Service management, Communication with other parts of the health care system, Communication with patients and carers, Transport interoperability, Innovation and performance  Administrative interoperability, Information and eHealth services, | [1, 11, 22-24, 30, 37-41, 59] |
|  | Internal interoperability | The extent to which the various information systems within the hospital are integrated and share information to enable coordination and cooperation | Integration optimization & innovation, Organizational layer of cooperation | [11, 30, 38, 39, 59] |
|  | Semantic interoperability | The extent to which the information exchanged between digital systems and applications is understandable to each, even if it was not the originally intended recipient. | Healthcare APIs, Semantic interoperability | [1, 21, 25, 34, 41] |
|  | Syntactic interoperability | The extent to which technical standards have been defined to enable the consistent, effective, and efficient integration of systems and services | Syntax interoperability, Technical layer of cooperation, Technical interoperability | [25, 30, 34, 41] |
| Strategy | Strategic adaptability | The extent to which the organization can adapt to strategic changes, whether internal or external | Competition, Population density, Readiness to change, Strategy | [11, 16, 24, 32, 43] |
|  | Strategic alignment | The extent to which the digital strategy is aligned with other strategies in the organization | Strategy, Digital strategy and literacy, Strategic alignment, People strategy  Strategic fit, Process strategy | [1, 11, 12, 30, 42] |
|  | Strategic focus | The extent to which the strategy of the organization focuses on one or more of the following: systematic evaluation of measurable goals and outcomes, removing inhibitors, quality, safety, sustainability, and cost-effectiveness | EA capability assessment, Linking strategy to value activities, Strategy, Quality strategy, Removal of inhibitors, Coverage, Assessment, Evaluation methods, Quality measures  Impact, | [21, 24, 26-28, 30, 32, 38] |
| Data Analytics | Descriptive analytics | The extent to which historical data facilitates effective decision making through analysis and reporting | Analytics and reporting, Analytics performance benchmarks, Data analysis | [1, 11, 16, 21, 40] |
|  | Predictive analytics | The extent to which business and clinical intelligence enables predictive decision making | Business and clinical intelligence, Learning health system, Proactive opportunity discovery, Population approach | [11, 24, 40] |
| Patient-centered care | Patient empowerment | The extent to which patients are encouraged to actively participate in their health decisions, and have access to information and health data | Citizen empowerment | [24] |
|  | Patient focus | The extent to which patients, caregivers and families are involved, and their input valued when designing new products | Customer focus | [25, 31] |
| Ref: Reference | | | | |
