# Supplementary material for: Synthesizing Dimensions of Digital Maturity in Hospitals: Systematic Review
Source: J Med Internet Res. 2022 Mar 30;24(3):e32994. doi: 10.2196/32994 (PMC9008527; doi:10.2196/32994)
Supplement: Multimedia Appendix 3 [file jmir_v24i3e32994_app3.docx]

**Multimedia Appendix 3.** Mapping of Dimensions to Maturity Models.

| **Maturity Model (MM)** | **Governance & Management** | **IT Capability** | **People, Skills & Behaviors** | **Interoperability** | **Strategy** | **Data Analytics** | **Patient-centered care** | **Reference** |
| --- | --- | --- | --- | --- | --- | --- | --- | --- |
| PACS MM (PMM) | X |  |  | X |  |  |  | [59] |
| extended PMM | X |  |  | X | X |  |  | [42] |
| MMEI | X |  |  | X |  |  |  | [34] |
| HIMSS Health Usability MM | X | X | X |  |  |  |  | [33] |
| PCMM | X |  | X |  |  |  |  | [51] |
| eCPR MM |  | X |  | X |  |  |  | [41] |
| QMS & ISO 10014 Standard | X | X |  |  |  |  | X | [31] |
| HRHCM | X | X | X |  | X |  |  | [26-28] |
| H-BIT |  | X |  |  | X |  |  | [30] |
| HSRM | X | X | X |  | X |  |  | [30] |
| HCMM |  |  |  | X |  |  |  | [30] |
| Patient-centric Framework |  | X | X | X |  |  | X | [25] |
| HC Game MM | X |  |  |  | X |  |  | [32] |
| DMA | X | X |  | X | X | X |  | [1] |
| HIMSS EMRAM | X | X | X | X | X | X |  | [11, 35, 43] |
| SCIROCCO’s B3-MM | X |  | X | X | X | X | X | [24] |
| IS4H-MM | X |  | X | X |  |  |  | [23] |
| HISMM | X | X | X | X | X | X |  | [16, 21] |
| Infrastructure MM - Australia | X | X |  | X |  |  |  | [37] |
| Interoperability MM – Greece |  |  |  | X |  |  |  | [39] |
| CDS MM | X | X |  | X |  | X |  | [40] |
| CDMI | X | X |  |  | X |  |  | [12, 22] |
| Patient RTF Framework |  | X | X | X | X |  |  | [38] |
| Digital excellence assessment | X | X | X | X | X |  |  | [11] |
| DMI | X | X |  |  | X | X |  | [11] |
| HITS Framework & SAFER guide | X | X | X | X |  |  |  | [22] |
| PCDHc | X |  | X |  |  |  |  | [29] |
