# Supplementary material for: Synthesizing Dimensions of Digital Maturity in Hospitals: Systematic Review
Source: J Med Internet Res. 2022 Mar 30;24(3):e32994. doi: 10.2196/32994 (PMC9008527; doi:10.2196/32994)
Supplement: Multimedia Appendix 4 [file jmir_v24i3e32994_app4.docx]

**Multimedia Appendix 4.** Method Applied for Validated Maturity Model.

| **Maturity Model (MM)** | **Method Applied** | **Findings** | **Ref^b^** |
| --- | --- | --- | --- |
| MMEI | Case study using an evaluation sheet applied to one of the hospitals involved in the eSanté-Cara project in Luxembourg | Case study hospital achieved a Level 2 maturity of interoperability  Did not examine maturity on outcomes related to the quadruple aims. | [34] |
| PCMM | Cross-sectional case study using questionnaires in medical record departments of government hospitals | No significant relevance between organizational maturity and the skills and capabilities of medical record practitioners was found  Did not examine maturity on outcomes related to the quadruple aims. | [51] |
| QMS & ISO 10014 Standard | Cross-sectional case study using questionnaires among hospital administrators and quality experts in hospitals with over 200 beds | No significant relevance between the organizational maturity of hospitals at different levels of implementing quality management systems was found  Did not examine maturity on outcomes related to the quadruple aims | [31] |
| HRHCM | 1. Analysis of 3232 patient safety reports and 67 RCA (root cause analysis) reports compared against the HRHCM  2. Survey data collected from 46 children’s hospitals  3. Secondary analysis of semi-structured interviews from a diverse and patient safety focused cross-section of study participants at 6 US Department of Veterans Affairs hospitals. | 1. Patient misidentification occurs both in inpatient and outpatient settings. Specific care areas indicated good safety culture. However, the focus was not on examining the outcomes resulting from HRHCM rather it was on identifying the root causes of adverse events and close calls.  2. Most participating organizations reported advancing or approaching high reliability practice in their hospitals. Did not examine the effect of maturity on outcomes related to the quadruple aims.  3. Findings suggest good content validity of the HRHCM in all 6 hospitals. However, a difference between hospitals was evident in the representation of the components of the model, with most hospitals being able to be evaluated at a specific maturity level. Ensured patient safety was embedded within the HRHCM, but did not explicitly examine the relationship between HRHCM levels with outcomes related to quadruple aims. | [26-28] |
| HC Game MM | Case study of the implementation of game at two university sites, providing a safe learning environment for emergency and elder care. | Findings suggest that organizational maturity is an influencing factor for the adoption of games development.  Did not examine maturity on outcomes related to the quadruple aims | [32] |
| DMA | Analysis of assessment questions targeting managers and front-line service users across English healthcare regions | Findings suggest that high levels of digital maturity and interoperability between organizations in a health service are underlying requirements for digital versatility and innovation  Did not examine maturity on outcomes related to the quadruple aims | [1] |
| HIMSS EMRAM | 1. Survey data collected at 600 Turkish hospitals to measure adoption rates and EHR use  2. Multivariate regression analysis of survey data collected from 73 hospitals with a valid EMRAM score and associated Dutch Surgical Colorectal Audit patients in the registry database | 1. Findings suggest that smaller hospitals adopt certain EHR functions better than larger hospitals  2. Findings suggest that for the entire group of hospitals there is a significant association between a higher EMRAM score and shorter LOS (Length of Stay) after colorectal cancer surgery. | [11, 35, 43] |
| SCIROCCO B3-MM | A three-round Delphi study was conducted to test the relevance of the dimensions, maturity indicators and assessment scales used | Satisfactory content validity of the B3-MM dimensions, maturity indicators and assessment scales established.  Did not examine maturity on outcomes related to the quadruple aims. | [24] |
| IS4H-MM | Rapid assessment to map NISH policy and develop a plan to strengthen and update IS4H-MM. Subsequently, a maturity assessment was performed to evaluate current implementation and determine next steps | The approval of NISH policy and adaption of IS4H-MM and subsequent assessment has positioned the territory as a leader and effective participant in the region for digital maturity  Did not examine maturity on outcomes related to the quadruple aims | [23] |
| Inter-operability MM | Team assessment of inpatient admission services in one public hospital and compared to assessments of 13 additional healthcare services. The interoperability maturity of the 14 healthcare services was compared to the maturity evaluation of 67 digital public services | Findings suggest that healthcare digital public services portray higher interoperability maturity than other public sectors in Greece  Did not examine maturity on outcomes related to the quadruple aims | [39] |
| CDS MM | CDS MM was developed through discussions with healthcare leaders from 80 organizations, an iterative model development by four clinical informaticists, and subsequent review with 19 healthcare organizations. Additionally, three case studies completed a self-assessment of the model at their healthcare organizations | Review of the model by 19 healthcare organizations confirmed the accuracy of its capabilities. The adoption of CDS in the three case study organizations was considered to be on the lower scale with only partial adoption of capabilities evident.  Did not examine maturity on outcomes related to the quadruple aims | [40] |
| CDMI | 1. Analysis of 130 healthcare regulator inspection reports and organizational digital maturity scores  2. Retrospective analysis of administrative data from 136 hospitals in England. Multivariable regression with 12 institutional covariates was used to examine the relationship between digital maturity and five clinical outcome measures | 1. Findings suggest a relationship between the positive use of health IT and overall organizational quality, although all organizations had significantly more references to the negative aspects of health IT  2. No significant relationship between organizational digital maturity and two outcome measures with a significant relationship between organizational digital maturity and risk-adjusted long length of stay and harm-free care | [12, 22] |
| Patient RTF Framework | Analysis of 13 studies that used digital systems to collect RTF | Evidence in the studies demonstrates that staff and patients are willing to engage in real-time feedback, but that the interoperability with other systems poses an issue overall  Did not specifically examine the level of maturity in relation to the quadruple aims. | [38] |
| HITS Framework & SAFER guide | Secondary analysis of 130 healthcare regulator inspection reports and organizational digital maturity scores | Findings suggest a relationship between the positive use of health IT and overall organizational quality, although all organizations had significantly more references to the negative aspects of health IT | [22] |
| ^a^This appendix includes only those papers in Appendix 2 that were identified as being applied/validated.  ^b^Ref: Reference | | | |
